# Challenges and Opportunities in Targeting the Complex Pancreatic Tumor Microenvironment

**DOI:** 10.1200/OA-24-00050

**Published:** 2024-12-18

**Authors:** Jennifer M. Finan, Yifei Guo, Shaun M. Goodyear, Jonathan R. Brody

**Affiliations:** ^1^ Department of Surgery, Oregon Health & Science University, Portland, OR; ^2^ Department of Cell, Developmental, and Cancer Biology, Oregon Health & Science University, Portland, OR; ^3^ Brenden-Colson Center for Pancreatic Care, Oregon Health & Science University, Portland, OR; ^4^ Knight Cancer Institute, Oregon Health & Science University, Portland, OR; ^5^ Division of Hematology and Oncology, School of Medicine, Oregon Health & Science University, Portland, OR

## Abstract

Pancreatic ductal adenocarcinoma (PDAC) is the third leading cause of cancer-related deaths with a 5-year survival rate of 13%. Surgical resection remains the only curative option as systemic therapies offer limited benefit. Poor response to chemotherapy and immunotherapy is due, in part, to the dense stroma and heterogeneous tumor microenvironment (TME). Opportunities to target the PDAC stroma may increase the effectiveness of existing or novel therapies. Current strategies targeting the stromal compartment within the PDAC TME primarily focus on degrading extracellular matrix or inhibiting stromal cell activity, angiogenesis, or hypoxic responses. In addition, extensive work has attempted to use immune targeting strategies to improve clinical outcomes. Preclinically, these strategies show promise, especially with the ability to alter the tumor ecosystem; however, when translated to the clinic, most of these trials have failed to improve overall patient outcomes. In this review, we catalog the heterogenous elements of the TME and discuss the potential of combination therapies that target the heterogeneity observed in the TME between patients and how molecular stratification could improve responses to targeted and combination therapies.

## INTRODUCTION

Pancreatic ductal adenocarcinoma (PDAC) tumors resemble an ecosystem, with up to 80% of the mass consisting of nontumor stromal cells and extracellular matrix (ECM).^1^ The PDAC ecosystem consists of matrix-producing cells, endothelial cells (ECs), immune cells, and noncellular components. The dense stroma and immunosuppressive nature of the tumor microenvironment (TME) may be the main reason for poor therapeutic responses observed in patients with PDAC. For example, TME components express immunosuppressive molecules, which promote extensive fibrosis, or desmoplasia, through increased ECM and cytokine secretion, which is associated with chemoresistance.^2^

The role of PDAC stroma is complicated as these heterogeneous elements can be both tumor-restrictive and tumor-promoting, underscoring the ambiguity in labeling the stroma as simply good or bad for tumor progression.^3^ Rather, different stromal functions likely depend on when and where it is evaluated in the tumorigenic process (eg, precursor lesions, PDAC). Therefore, while eliminating specific stromal components may be effective at one point during tumorigenesis, inhibiting the same element(s) at another point during tumor development could promote progression. In addition, while inhibiting specific pathways in a cell type may be beneficial, off-target inhibition of the same pathways in other cells could lead to tumor progression.

Herein, we define the elements of the heterogenous PDAC TME and then outline the different approaches to target the TME. We attempt to provide a state of the union of preclinical and clinical therapeutic strategies for PDAC that have shown promising evidence in combination strategies, from degradation of the ECM to immune checkpoint blockade (ICB) therapies.

## CELLULAR COMPOSITION OF THE PDAC TME

### Nonimmune Stromal Cells

#### Cancer-Associated Fibroblasts

Cancer-associated fibroblasts (CAFs) are a prominent cell type within the TME, which are heterogeneous in both origin and function.^4,5^ Resident mesenchymal cells, pancreatic stellate cells (PSCs), are responsible for maintaining ECM homeostasis in the normal pancreas. During tumorigenesis, PSCs are activated by increased reactive oxygen species, cytokines (eg, interleukin [IL]-6, IL-10, sonic hedgehog [Shh], tumor necrosis factor [TNF]-α), and growth factors (eg, transforming growth factor [TGF]-β), inducing their differentiation into CAFs. PSCs were once thought to be the primary origin of CAFs; however, we now understand that only 10%-15% of CAFs are derived from PSCs.^5^ The remainder of CAFs are now thought to originate from resident fibroblasts, ECs, bone-marrow derived macrophages, mesothelial cells, or mesenchymal stem cells.^6,7^ It remains an active area of investigation whether a CAF's origin determines its function and whether CAF subtypes are influenced by their origin.

The best characterized CAF subtypes are αSMA^high^, ECM-producing myofibroblastic CAFs (myCAFs), and IL-6^high^ inflammatory CAFs (iCAFs). They express enzymes required for hyaluronan production and secretion of inflammatory cytokines that support immunosuppression and chemoresistance.^8-11^ In vivo modeling, however, shows that CAF subpopulations are not static as iCAFs can transform into myCAFs.^12^ Another CAF subpopulation of antigen-presenting CAFs (apCAFs) is also capable of transforming into myCAFs, but their activation conditions remain unclear. These apCAFs lack the necessary costimulatory molecules (CD80, CD86, and CD40) for inducing CD4^+^ T-cell clonal proliferation, suggesting a distinct role from canonical antigen-presenting cells (APCs).^10^ Overall, CAFs are diverse, emphasizing the challenge of targeting subpopulations and highlighting the need for nuanced therapeutic strategies when targeting the PDAC TME.

#### Endothelial Cells

Angiogenesis is a long-described hallmark of PDAC that supports tumor growth through increased vessel formation and/or increased metastatic spread via dysfunctional leaky vasculature.^13^ Environmental cues including hypoxia and various growth factors (vascular EC growth factor [VEGF], fibroblast growth factor [FGF]) increase EC recruitment and angiogenesis.^14^ Within PDAC tumors, ECs compose tumor blood and lymphatic vasculature, which can be both tumor-promoting and tumor-restrictive.^14^ Hypovascularization in PDAC because of high interstitial pressures and dense stroma limits both immune surveillance and drug delivery.^15^ Conversely, increased vascular signaling improves prognosis and is linked to higher anticancer immunity, underscoring the importance of practical considerations in targeting vessel formation, as it may hinder other immune-mediated therapeutic approaches.^16-18^

### Immune Cells

#### Dendritic Cells

Dendritic cells (DCs) are relatively rare within the PDAC TME.^3,19^ DCs are APCs that display foreign antigens to T-helper and cytotoxic T-lymphocytes (CTLs) to elicit their activation. PDAC tumors suppress DC activity through secretion of cytokines (TGF-β, IL-10, and IL-6).^20^ As such, increased circulating DCs correlate with a better prognosis in patients with PDAC.^21^ Considering the various functions and potential subtypes of DCs, therapeutic strategies that improve the infiltration and activity of DCs could aid immunotherapy for PDAC.

#### CD8^+^ T Cells

CD8^+^ T cells or CTLs possess the potential to selectively eliminate PDAC cells through the release of cytotoxins. T-cell activation is regulated by immune checkpoint proteins such as PD-1, cytotoxic T lymphocyte antigen-4 (CTLA-4), lymphocyte activation gene-3, T-cell immunoglobulin and mucin domain–containing-3 (TIM-3), and T-cell immunoreceptor with immunoglobulin and ITIM domain (TIGIT).^22^ Understanding the role of these proteins in regulating CD8^+^ T cell–mediated killing of tumor cells has been key to the development of ICB strategies.^23^

#### CD4^+^ T Cells

CD4^+^ T helper (Th) cells often are classified into Th1, Th2, Th17, and T_reg_-cell subtypes, recognize neoantigens, and coordinate with other immune cells.^24^ In patients with PDAC, a shift from Th1 to Th2 denotes poor prognosis. Th1 cell activation of CTLs and macrophages to facilitate antitumor immunity is supplanted by Th2 cell–mediated fibrogenesis and stimulation of tumor-supporting macrophages.^25^ The role of Th17 cells in tumor immunity is controversial as they can be both tumor-restrictive and tumor-promoting.^26^ T_reg_ cells are recruited to tumors via secreted factors such as C-C motif ligand (CCL)2 and CCL5,^27,28^ where they induce CTL suppression and cytolysis via expressed and secreted molecules (eg, IL-10, TGF-β, CTLA-4, granzyme B). Perturbation of T_reg_ cells in an established PDAC mouse model showed improved antitumor responses.^29^ High levels of T_reg_-cell infiltration also correlate with poor prognosis in patients, and thus, therapies inhibiting T_reg_ cells to boost Th1-mediated antitumor immunity are hypothesized to provide clinical benefit.^30^

#### Macrophages

Macrophages are innate immune cells capable of engulfing pathogens, presenting antigens to CTLs, and communicating with other immune cells. Tumor-associated macrophages (TAMs), a macrophage subtype, comprise most of the leukocytes in the PDAC TME and exert immune suppressive functions. TAMs originate from both adult hematopoietic stem cells and embryonically derived pancreatic resident macrophages capable of self-renewal.^31^ Hematopoietic stem-cell–derived C-C motif receptor (CCR)2^+^ monocytes are subsequently recruited to the TME via high expression of CCL2,^32^ where they induce an immunosuppressive response through the secretion of cytokines and chemokines. TAM secretion of TGF-β and IL-10, for instance, recruits T_reg_ cells and inhibits CTL activity.^33^ TAM surface expression of PD-L1 may also induce T-cell cytolysis.^34^ Embryonic-derived TAMs, however, can activate PSCs to stimulate ECM secretion.^35,36^

## THERAPEUTIC APPROACHES TARGETING THE PDAC TME

### Degradation of the ECM

**FIG 1.**
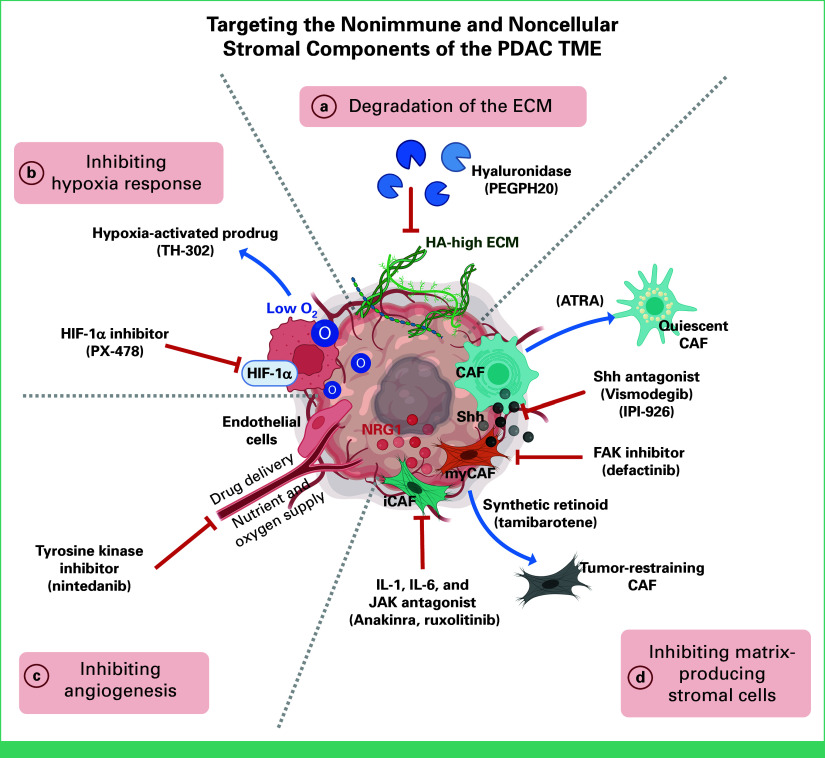
Targeting the nonimmune stromal compartment of the PDAC TME. A summary of the current stromal-targeting therapies in PDAC, including (a) degradation of HA in ECM with hyaluronidase; (b) inhibiting hypoxia response in tumor cells with HIF-1α inhibitor and taking advantage of hypoxia with the use of hypoxia-activated prodrugs; (c) inhibiting angiogenesis with tyrosine kinase inhibitors; (d) targeting matrix-producing stromal cells by inhibiting the activity of PSCs with CXCR4 inhibitor, inhibiting PSC-derived Shh with antagonist, and inhibiting myCAFs with FAK inhibitors and iCAFs with IL-1, IL-6, and JAK antagonist. Alternatively, matrix-producing stromal cells are targeted to convert from CAFs to quiescent PSCs with ATRA and from myCAFs to tumor-restraining CAFs with synthetic retinoids. ATRA, all-trans retinoic acid; CAF, cancer-associated fibroblast; CXCR4, C-X-C chemokine receptor type 4; ECM, extracellular matrix; FAK, focal adhesion kinase; HA, hyaluronic acid; HIF-1α, hypoxia-inducible factor-1 alpha; iCAF, inflammatory cancer-associated fibroblast; IL-1, interleukin-1; IL-6, interleukin-6; JAK, Janus kinase; myCAF, myofibroblastic cancer-associated fibroblast; PDAC, pancreatic adenocarcinoma; PSC, pancreatic stellate cell; Shh, sonic hedgehog; TME, tumor microenvironment.

Increased interstitial fluid pressure and impaired blood flow are hallmarks of the PDAC TME that contributes to immune evasion and chemoresistance, and thus, targeting ECM components is thought to alleviate these stresses (Fig 1).^37^ However, clinical trials evaluating ECM degradation to bolster delivery of chemotherapy or improve antitumor immunity have been unsuccessful. Degradation of hyaluronic acid using pegylated recombinant human hyaluronidase (PEGPH20), for example, failed to show benefit with gemcitabine (GEM) or folinic acid [leucovorin], fluorouracil [5-FU], irinotecan, and oxaliplatin (FOLFIRINOX)^38^ or with anti–PD-L1 therapy.^39-41^ Salvaging the clinical utility of PEGPH20 likely requires additional targeting of the TME as preclinical studies show that PEGPH20 improves anti–PD-1 sensitivity with the additional inhibition of intracellular focal adhesion kinase (FAK)^39^ or CXCR4/CXCL12 signaling in stromal cells (discussed below).

Another ECM-targeting strategy is pamrevlumab, a monoclonal antibody that inhibits the activity of connective tissue growth factor, whose overabundance is a primary driver of fibrosis. Unfortunately, two late-stage trials investigating pamrevlumab alone or in combination with GEM-nab or FOLFIRINOX did not meet the primary end points of overall survival (OS) and the manufacturer, FibroGen, has terminated all research and development using pamrevlumab.^42^ Future advances in the field likely require tailored combination of stroma-targeted therapies that can modulate many aspects of the heterogenous TME.

### Inhibiting Stromal Cell Function and Activity

Activated PSCs and CAFs contribute to the treatment-resistant PDAC TME via expression of surface molecules and secretion of matrix proteins, cytokines, chemokines, and metabolites.^43^ Numerous inhibitors targeting Shh, TGF-β, FAK, neuregulin (NRG)1, and all-trans retinoic acid (ATRA) are being leveraged to promote PSC and CAF quiescence. Shh inhibitors aim to inhibit CAF ECM production, thereby decreasing the desmoplastic TME; however, their efficacy in preclinical models has not improved outcomes for patients with PDAC.^9,44^ No progression-free survival (PFS) or OS benefit was seen in trials giving patients with metastatic PDAC (mPDAC) GEM with Shh antagonists, vismodegib or IPI-926, and in combination with FOLFIRINOX, IPI-926 increased disease progression.^45-47^ Combining GEM-nab with smoothened inhibitor, LDE225, had minimal median PFS or OS improvements although magnetic resonance imaging assess showed increases in tumor perfusion, suggesting that LDE225 reduces PSC/CAF populations.^48,49^ These poor outcomes may reflect that Shh inhibition primarily affects myCAFs, where initial benefits are soon overcome by increased iCAFs that promote disease progression.^50^ Interestingly, an emerging hedgehog inhibitor, NLM-001, administered before a combination of GEM-nab and anti–CTLA-4 (zalifrelimab) showed promising responses as a second-line treatment in patients with mPDAC.^51^ Preliminary evaluation of paired tumor biopsies from one patient showed reductions in CAFs, T_reg_, and macrophages after 21 days of treatment.

Another strategy to reduce PSC and CAF activation uses retinoid-acid based treatments, which supply CAFs with an active metabolite of vitamin A, suppressing CAF contractility and mechanosensing to decrease ECM remodeling.^52^ ATRA in combination with GEM-nab was well tolerated in patients with PDAC, showing reduced neurotoxicity attributed to nab-paclitaxel,^53^ and a larger efficacy trial is underway (ClinicalTrials.gov identifier: NCT04241276). Tamibarotene is a synthetic retinoid, which in PDAC mouse models increases tumor-restraining Meflin^+^ CAFs, which was associated with decreased collagen deposition, increased tumor vasculature, and enhanced GEM sensitivity.^54^ Tamibarotene is currently being investigated in combination with GEM-nab in patients with advanced PDAC.^55^

TGF-β overexpression has a multitude of downstream effects on the TME as it alters cancer cell metabolism, promotes epithelial-to-mesenchymal transition (EMT), increases myCAF activation, and supports infiltration of immune suppressive cells.^56-58^ Inhibiting TGF-β signaling partially attenuates tumor cell growth by altering CAF and immune cell dynamics^59-62^; however, the complexity of inhibiting TGF-β signaling is reflected by unsuccessful trials and discontinued development of several drug candidates (Table 1).^153,154^ These limitations may partially be attributed to targeting myCAF function without abating the immunosuppressive iCAFs.

**TABLE 1.** Completed and Ongoing Clinical Trails on Patients with PDAC Grouped by Targeting Strategy

Target	Agent	Combination Regimen	Phase	Status	Outcomes	Clinical Trial
Hyaluronan	Pegvorhyaluronidase alfa PEGPH20	GEM-nab, dexamethasone, or enoxaparin	II	Completed	HA^high^ tumor response increased from 31% to 45% mPFS: PEGPH20 + chemo (6.0 months) *v* chemotherapy alone (5.3 months; HR, 0.73 [95% CI, 0.53 to 1.00]; *P* = .049) mPFS in HA^high^ tumors: PEGPH20 + chemotherapy (9.2 months) *v* chemotherapy alone (5.2 months, HR, 0.51 [95% CI, 0.26 to 1.00]; *P* = .048)	NCT01839487 ^63^
		FOLFIRINOX	I/II	Active, not recruiting	PEGP20 + FOLFIRINOX increased toxicity and decreased the treatment period and thus did not extend survival	NCT01959139 ^64^
		GEM-nab	III	Terminated	OS: PEGPH20 + chemotherapy (11.2 months) *v* chemotherapy alone (11.5 months). mPFS: PEGPH20 + chemotherapy (7.1 months) *v* chemotherapy alone (7.1 months, HR, 0.97 [95% CI, 0.75 to 1.26]). ORR: PEGPH20 + chemotherapy (47%) *v* chemotherapy alone (36%, ORR ratio, 1.29 [95% CI, 1.03 to 1.63])	NCT02715804 ^65^
		Pembrolizumab	II	Unknown	Pembrolizumab + PEGPH20 did not increase PFS compared with historical data. mOS: 7.2 months (95% CI, 1.2 to 11.8), mPFS: 1.5 months (95% CI, 0.9 to 4.4) Best response: stable disease (n = 2, 25%) lasting 2.2 and 9 months, respectively No difference in CD8^+^ T-cell infiltration or PDL1 expression with OS or best overall response	NCT03634332 ^40^
		Pembrolizumab, GEM-nab	II	Withdrawn	Study withdrawn	NCT04045730
		Avelumab (PD-L1)	I	Terminated	No results posted	NCT03481920
		GEM	Ib	Completed	Promising clinical activity, especially in patients with HA^high^ tumors. mPFS: HA^high^ patients 219 days (95% CI, 159 to 276), HA^low^ patients 108 days (95% CI, 14 to 163) mOS: HA^high^ patients 395 days (95% CI, 210 to 578), HA^low^ patients 174 days (95% CI, 34 to 293)	NCT01453153 ^66^
		Pembrolizumab	II	Withdrawn	Study withdrawn	NCT04058964
	Recombinant human hyaluronidase (rHuPH20)	Nivolumab	I/II	Active, not recruiting	No results posted (CheckMate 8KX trial)	NCT03656718
CTGF	Pamrevlumab	GEM-nab	I/II	Completed	Pamrevlumab + GEM-nab *v* GEM-nab mPFS: 14.1 months *v* 11.6 months mOS: NR *v* 18.56 months	NCT02210559 ^42^
		GEM-nab or FOLFIRINOX	III	Active, not recruiting	Well tolerated. No clinical efficacy found	NCT03941093
		GEM-nab or FOLFIRINOX	II/III	Active, not recruiting	Did not meet primary end point for OS	NCT04229004
MMP9	Andecaliximab	Nivolumab	II		No additional benefit with andecaliximab ORR: andecaliximab + nivolumab = 10% (95% CI, 4 to 19), nivolumab = 7% (95% CI, 2 to 16)	NCT02864381 ^67^
LAIR1	NGM438	Pembrolizumab	I/Ib	Recruiting	No results posted (KEYNOTE-E20)	NCT05311618
Hedgehog	NLM-001	GEM-nab + zalifrelimab (CTLA4)	I/II	Active, not recruiting	n = 22 evaluable patients mPFS: 7.1 months ORR: 50% DCR: 95%	NCT04827953
SMOOTHENED	Saridegib (IPI-926)	GEM	Ib/II	Completed	Trial terminated. Patients receiving IPI-926 had a shorter median survival time and more rapid rate of disease progression compared with the placebo-containing arm	NCT01130142
		FOLFIRINOX	I	Completed	n = 15 response-evaluable patients ORR: 66.7%; mPFS: 8.4 months	NCT01383538 ^45^
	Vismodegib	GEM	I/II	Completed	Vismodegib + GEM-nab *v* GEM-nab DCR: 58% *v* 51%; mPFS: 4.0 months *v* 2.5 months; adj. HR = 0.83 (95% CI, 0.55 to 1.23) mOS: 6.9 months *v* 6.1 months	NCT01064622 ^47^
	Sonidegib (LDE225)	GEM-nab	I/II	Completed	n = 24 evaluable patients Three PR (13%), 14 SD (58%), seven PD (29%). mOS: 6.0 months (IQR, 3.9-8.1) mPFS: 4.0 months (IQR, 1.2-6.7)	NCT02358161 ^48^
CXCR4	Motixafortide (BL-8040)	Pembrolizumab with or without NALIRIFOX	IIa	Active, not recruiting	Motixafortide + pembrolizumab (N = 34) OS: 3.3 months ORR: 3.4% DCR: 34.5%; median duration of response = approximately 2.7 months Motixafortide + pembrolizumab + NALIRIFOX (N = 22) ORR: 32% DCR: 77%; median duration of response = 7.8 months	NCT02826486 ^68^
	MB1707			Withdrawn	No results posted	NCT05465590
	Plerixafor (AMD3100)		I	Terminated	Terminated because of slow accrual	NCT03277209
			I	Completed	No CR or PR (N = 23) 13 patients (57%) achieved SD, and 10 (43%) PD	NCT02179970 ^69^
CXCL12	Olaptesed pegol (NOX-A12)	Pembrolizumab	I	Completed	No objective responses, 25% achieved SD (N = 10 [1 CRC, 9 PC]). mPFS: 1.87 months mOS: 3.97 months	NCT03168139 ^70^
		Pembrolizumab + nanoliposomal irinotecan, or GEM-nab	II	Not yet recruiting	No results posted	NCT04901741
TGF-β	BCA101	Pembrolizumab	I	Recruiting	n = 15 (39%) evaluable patients at reporting PR: 3 of 11 (27%) evaluable patients (two in SCAC, one in HNSCC) DCR: 82% (9 of 11 patients) ORR (HNSCC expansion cohort): 44% (eight PR) CBR (PR + SD): 67%	NCT04429542 ^71-73^
	HCW9218		I/II	Active, not recruiting	N = 15 SD: 2 (13%)	NCT05304936 ^74^
			I	Active, not recruiting	Over 70% (five of seven) of patients with ovarian cancer showed stable disease	NCT05322408
	NIS793^a^	Spartalizumab	I	Completed	N = 120 ORR: 3% (four PR) SD: 28 (23%) of 120 patients PD: 71 (59%) of 120 patients	NCT02947165 ^75^
		Spartalizumab, FOLFIRINOX, Chemoradiation	I	Terminated	Novartis discontinued development because of insufficient efficacy	NCT05417386 ^76^
		Spartalizumab, GEM-nab	II	Terminated	Novartis discontinued development because of insufficient efficacy	NCT04390763 ^77^
		GEM-nab	III	Completed	No results posted	NCT04935359
	AVID200		I	Unknown	SD >12 weeks: two patients (one adenoid cystic carcinoma, one with breast carcinoma)	NCT03834662 ^78^
	SAR439459^a^	Cemiplimab (PD-1)	I	Terminated	Discontinued because of a lack of efficacy	NCT03192345 ^79^
	Livmoniplimab (ABBV-151)	Budigalimab (ABBV-181)	I	Recruiting	In the combination dose-escalation cohorts: four confirmed responses, one unconfirmed response, and four patients had SD (≥6 months) In anti–PD-1/PD-L1 relapsed/refractory UC EXP cohort: five confirmed responses, one unconfirmed response, and five SD In anti–PD-1/PD-L1–naïve HCC expansion cohort: five confirmed responses and three SD	NCT03821935 ^80^
	Dalutrafusp alfa (AGEN1423/GS-1423)		I	Terminated	Discontinued development N = 17 ORR: 4.8% (90% CI, 0.2 to 20.7) DCR: 38.1% (90% CI, 20.6 to 58.3)	NCT03954704 ^81^
	SRK-181	Pembrolizumab	I	Active, not recruiting	Well tolerated	NCT04291079 ^82^
	LY3200882	GEM-nab	I	Active, not recruiting	n = 12 patients with treatment-naïve advanced pancreatic cancer ORR: 50% (6 of 12) DCR: 75% (9 of 12), respectively Median duration of response: 4.0 months CA19-9 levels decreased by >50%: 8 of the 12 patients	NCT02937272 ^83^
	SH3051		I	Unknown	No results posted	NCT04423380
TGFβR1	PF-06952229^a^		I	Terminated	Enrollment termination not related to safety concerns	NCT03685591
	Galunisertib (LY2157299)^a^	Durvalumab	Ib	Completed	n = 32 response-evaluable patients One PR, seven SD, 15 PD (nine not evaluable) DCR: 25.0% mPFS: 1.87 months (95% CI, 1.58 to 3.09) mOS: 5.72 months (95% CI, 4.01 to 8.38)	NCT02734160
		GEM	I/II	Completed	Galunisertib + GEM *v* placebo + GEM OS: 10.9 months *v* 7.2 months mPFS: 4.11 (2.66 to 5.42) months *v* 2.86 (1.94 to 3.75) months ORR: 10.6% (5.4 to 18.1) *v* 3.8% (0.5 to 13.2)	NCT01373164
		GEM	I	Completed	N = 7; mPFS: 64 days	NCT02154646
	Vactosertib (TEW-7197)	FOLFOX	I/II	Unknown	n = 13 evaluable patients treated at established RP2D Three PR, five SD CBR: 61.5%; mPFS: 5.6 months (95% CI, 2.27 to 8.93)	NCT03666832
		nal-IRI/FL	Ib	Unknown	No results posted	NCT04258072
TGF-β/anti–PD-L1	Y101D (YM101)		I	Active, not recruiting	No results posted	NCT05028556
	Bintrafusp alfa^a^		I	Completed	n = 18 response-evaluable patients One CR, two PR (including one PC), six SD (including three PC), nine PD	NCT02517398 ^61^
	QLS31901		I	Unknown	No results posted	NCT04954456
	TST005		I	Terminated	Corporate decision	NCT04958434
TGFβRII/anti-PD-L1	PM8001		I	Active, not recruiting	n = 67 response-evaluable patients ORR: 10.4% (95% CI, 4.3 to 20.4) DCR: 53.7% (95% CI, 41.1 to 66.0)	ChiCTR2000033828^84^
	SHR-1701	GEM-nab	Ib/II	Unknown	n = 52 evaluable patients ORR: 36.5% (95% CI, 23.6 to 51.0) DCR: 80.8% (95% CI, 67.5 to 90.4)	NCT04624217
TGF-β/TIGIT	AK130		I	Completed	No results posted	NCT05653284
TGFβRI/VEGFR2	TU2218	Pembrolizumab	I/II	Recruiting	No results posted	NCT05204862 ^85^
TGFβ/VEGF	ZGGS18		I/II	Recruiting	N = 21 Safe and tolerable dose escalation	NCT05584800
TGFβ1, GARP	JYB1907		I	Not yet recruiting	No results posted	NCT05821595
FAK	VS-4718	GEM-nab	I	Terminated	Corporate decision	NCT02651727
	Defactinib		II	Recruiting	No results posted	NCT03727880 ^86^
		Pembrolizumab, GEM	I	Completed	n = 13 evaluable patients Seven SD	NCT02546531 ^87^
		SBRT	II	Recruiting	No results posted	NCT04331041
	GSK2256098	Trametinib	II	Completed	n = 11 evaluable patients One SD, 10 PD	NCT02428270 ^88^
IL-1R	Anakinra	mFOLFIRINOX	I	Unknown	mPFS: 5 months mOS: not been reached (median follow-up of 15 months [3-25]) 1-year OS: 75%	NCT02021422 ^89^
		GEM-nab		Completed	No efficacy results posted	NCT02550327 ^90^
	Sorafenib	GEM	III	Unknown	No improvement in PFS	NCT00541021 ^91^
IL-1β	Canakinumab	Spartalizumab, GEM-nab	III	Active, not recruiting	No results posted	NCT04229004 ^92^
			Ib	Active, not recruiting	No DLT for phase I	NCT04581343 ^93^
		Tislelizumab, GEM-nab	Ib	Recruiting	No efficacy results posted	NCT05984602
IL1RAP	Nadunolimab	FOLFIRINOX	I	Completed	No results posted	NCT04990037
		GEM-nab	I	Active, not recruiting	n = 33 PC-evaluable patients ORR: 27% CBR: 57.6%. mDoR: 6.5 months (range, 1.9-13.8) mPFS (per iRECIST): 7.8 months (95% CI, 5.2 to 10.2) mOS: 12.6 months (95% CI, not estimable) OS 1-year: 55%	NCT03267316 ^94^
JAK	Ruxolitinib	Capecitabine	II	Completed	mOS: 4.5 months with ruxolitinib + capecitabine *v* 4.3 months with placebo + capecitabine	NCT01423604 ^95^
		Capecitabine	III	Terminated	JANUS 1: ruxolitinib + capecitabine (n = 161) *v* placebo + capecitabine (n = 160) OS: 89.0 days *v* 93.0 days (HR, 0.969 [95% CI, 0.747 to 1.256]; *P* = .409) PFS: 43 days *v* 44 days (HR, 1.056 [95% CI, 0.827 to 1.348]; *P* = .666) ORR: 3.7% *v* 1.9% (odds ratio, 2.13 [95% CI, 0.44 to 13.48]) JANUS 2: ruxolitinib + capecitabine (n = 43) *v* placebo + capecitabine (n = 43) OS: 108 days *v* 149 days (HR, 1.584 [95% CI, 0.886 to 2.830]; *P* = .942) PFS: 61 days *v* 48 days (HR, 1.166 [95% CI, 0.687 to 1.978]; *P* = .720) ORR: 4.7% *v* 1.0% (odds ratio, 2.39 [95% CI, 0.11 to 162.0])	NCT02119663 ^96^
IL-6	Siltuximab	Spartalizumab (PDR001)	Ib/II	Completed	ORR = 0% (N = 14)	NCT04191421 ^97^
	Tocilizumab	GEM-nab	II	Completed	Tocilizumab + GEM-nab *v* GEM-nab OS at 6 months: 68.6% (95% CI, 56.3 to 78.1) *v* 62.0% (95% CI, 49.6 to 72.1; *P* = .41) mOS: 8.4 *v* 8.0 months (HR, 0.75 [95% CI, 0.54 to 1.05]; *P* = .10) mPFS: 5.6 *v* 5.5 months (HR, 0.85 [95% CI, 0.61 to 1.20]; *P* = .36) ORR: 37.1% (95% CI, 25.9 to 49.5) *v* 35.2% (95% CI, 24.2 to 47.5)	NCT02767557 ^98^
LIF	AZD0171		I	Completed	No objective responses mPFS: 5.9 weeks	NCT03490669 ^99^
		Durvalumab	II	Active, not recruiting	No results posted	NCT04999969
TKI	Nintedanib	GEM	I/II	Terminated	Terminated because of lack of future funding	NCT02902484
	PLX3397 (pexidartinib)	Durvalumab	I	Completed	n = 47 response-evaluable patients (n = 24 CRC, n = 23 PC) 1 PR, 7 SD, 39 PD	NCT02777710
HER2/3	Zenocutuzumab (MCLA-128)		II	Recruiting	n = 71 evaluable patients ORR: 34% (90% CI, 25 to 44), including responses in 14 NSCLC, seven PC, two breast cancer, and one cholangiocarcinoma. mDOR: 9.1 months (95% CI, 5.2 to 12.0) DOR at 6 months: 70%	NCT02912949 ^100^
	Seribantumab		II	Completed	No results posted	NCT04790695
			II	Active, not recruiting	n = 10 evaluable patients ORR: 30% DCR: 90% (one CR, two PR, six SD, one PD)	NCT04383210
	HMBD-001		I/II	Recruiting	No results posted	NCT05057013 ^101^
RAR	ATRA	GEM-nab	Ib	Completed	ATRA was tolerable and safe. ATRA decreased frequency and intensity of neurotoxicity attributed to nab-paclitaxel Upregulation of pentraxin 3 (PTX3) in PSC cells with <6 month ATRA, but not ATRA >6 months, suggesting limiting the duration of ATRA to 6 months DW-MRI shows increased diffusion coefficient after 1 month ATRA; suggesting stromal modulation	NCT03307148 ^53^
		GEM-nab	IIb	Active, not recruiting	Not yet recruiting	NCT04241276
	Tamibarotene	GEM-nab	I/II	Recruiting	No results posted	NCT05064618 ^55^
VEGFA	Bevacizumab	FU, NANT-008, leucovorin, and oxaliplatin	I/II	Withdrawn	Withdrawn because of no enrollment	NCT03127124
		Erlotinib, RT	I/II	Completed	No results posted	NCT00735306
		Capecitabine, RT	I	Completed	No results posted	NCT00047710
		GEM, FU	II	Completed	PFS at 6 months: 49% ORR: 30% (12 PR, 18 SD, 10 PD)	NCT00417976
		Erlotinib, capecitabine, RT	I	Completed	No results posted	NCT00614653
		GEM, RT	II	Completed	n = 12 evaluable patients: 10 SD, 2 PD	NCT00460174 ^102^
		Erlotinib	II	Completed	n = 36 evaluable patients: one PR, seven SD Median time to progression: 40 (95% CI, 35 to 41) days mOS: 102 (95% CI, 74 to 117) days	NCT00365144 ^103^
		GEM, FU, oxaliplatin, radiation	II	Completed	No results posted	NCT00602602
		GEM	II	Completed	OS at 6 months: 77% mOS: 8.8 months mPFS: 5.4 months	NCT00126633 ^104^
		GEM	III	Completed	GEM + bevacizumab (n = 302) *v* GEM + placebo (n = 300) mOS: 5.8 months (95% CI, 4.9 to 6.6) *v* 5.9 months (95% CI, 5.1 to 6.9) (*P* = .95) mPFS: 3.8 months (95% CI, 3.4 to 4.0 months) *v* 2.9 months (95% CI, 2.4 to 3.7 months; *P* = .075) ORR: 13% (1% CR, 12% PR) *v* 10% (1% CR, 9% PR)	NCT00088894 ^105^
		Capecitabine + RT followed by GEM	II	Completed	n = 82 evaluable patients; mOS: 11.9 months (95% CI, 9.9 to 14.0) mPFS: 8.6 months (95% CI, 6.9 to 10.5) Response: 50 SD (61%), 21 PD (26%)	NCT00114179
VEGFR	Axitinib	GEM	III	Completed	GEM + axitinib (n = 314) *v* GEM + placebo (n = 316) mOS: 8.5 months (95% CI, 6.9 to 9.5) *v* 8.3 months (95% CI, 6.9 to 10.3; HR, 1.014 [95% CI, 0.786 to 1.309]; *P* = .5436) mPFS: 4.4 months *v* 4.4 months (HR, 1.006 [95% CI, 0.779 to 1.298]; *P* = .5203) ORR: 5% (95% CI, 2.5 to 8.3) *v* 2% (95% CI, 0.4 to 4.0)	NCT00471146 ^106^
DNA alkylator (HIF1α)	Evofosfamide (TH-302)	GEM	III	Completed	Evofosfamide + GEM (n = 346) *v* placebo + GEM (n = 347) mOS: 8.9 months *v* 7.6 months (HR, 0.84 [95% CI, 0.71 to 1.01], *P* = .059) mPFS: 5.5 months *v* 3.7 months (HR, 0.77 [95% CI, 0.65 to 0.92], *P* = .004) ORR: 15% *v* 9% (odds ratio, 1.90 [95% CI, 1.16 to 3.12], *P* = .009)	NCT01746979 ^107^
		GEM	II	Completed	Evofosfamide (240 mg/m^2^; 30 minutes on days 1, 8, and 15 of every 28-day cycle) + GEM (n = 71) *v* GEM (n = 69) mPFS: 5.6 months *v* 3.6 months (HR, 0.61 [95% CI, 0.43 to 0.87]; *P* = .005) mOS: 8.7 months *v* 6.9 months (HR, 0.95 [95% CI, 0.67 to 1.34], *P* = .77) Evofosfamide (340 mg/m^2^; 30 minutes on days 1, 8, and 15 of every 28-day cycle) + GEM (n = 74) *v* GEM (n = 69) mPFS: 6.0 months *v* 3.6 months (HR, 0.59 [95% CI, 0.40 to 0.87], *P* = .008) mOS: 9.2 months *v* 6.9 months (HR, 0.86 [95% CI, 0.61 to 1.21], *P* = .39)	NCT01144455 ^108^
		Sunitinib	II	Completed	Responses: one CR, two PR, 11 SD; mPFS: 10.4 months (95% CI, 2.6 to 18.0)	NCT02402062 ^109^
CD40	Sotigalimab (PYX-107, APX005M)	Nivolumab, GEM-nab	I/II	Completed	n = 105 response-evaluable patients Sotiga/nivo/chemo group (N = 35, 1-year OS = 41.3%)	NCT03214250
	Selicrelumab (CP-870,893)	GEM	I	Completed	n = 21 evaluable patients ORR: 19% (four PR, 11 SD) mPFS: 5.2 months (95% CI, 1.9 to 7.4) mOS: 8.4 months (95% CI, 5.3 to 11.8)	NCT00711191 ^110^
	Mitazalimab	mFOLFIRINOX	II	Active, not recruiting	n = 23 evaluable patients ORR = 52.2% (12 PR) DCR = 91.3% (12 PR, eight SD)	NCT04888312 ^111^
	SEA-CD40	Pembrolizumab, GEM-nab	I	Terminated	No efficacy results Trial terminated because of portfolio prioritization	NCT02376699
	NG-350A	Ipilimumab, GEM-nab	I	Active, not recruiting	No results posted	NCT04787991
	CDX-1140	CDX-301	I	Terminated	Corporate decision	NCT04536077
	YH003	Toripalimab	I/II	Completed	n = 16 evaluable patients one CR, one PR, three SD	NCT04481009 ^112^
CD40	CDX-1140	Odetiglucan	Ib	Terminated	Corporate decision	NCT05484011 ^82^
CD137	Urelumab (BMS-663513)	GVAX, nivolumab	I/II	Recruiting	GVAX alone (arm A), GVAX + nivolumab (arm B), GVAX + nivolumab + urelumab (arm C) DFS: GVAX alone *v* GVAX + nivolumab (HR, 1.09 [95% CI, 0.50 to 2.40], *P* = .829) DFS: GVAX alone *v* GVAX + nivolumab + urelumab (HR, 0.55 [95% CI, 0.21 to 1.49], *P* = .242) DFS: GVAX + nivolumab *v* GVAX + nivolumab + urelumab (HR, 0.51 [95% CI, 0.19 to 1.35], *P* = .173)	NCT02451982 ^113^
TRL8	Motolimod (VTX-2337)	Cyclophosphamide	I	Terminated	Corporate decision	NCT02650635
		Cisplatin or carboplatin + FU + cetuximab	Ib/II	Completed	n = 13 evaluable patients ORR = 15% DCR = 54% (two PR, five SD)	NCT01836029 ^114^
TLR9	Lefitolimod (MGN1703)	Ipilimumab	I	Active, not recruiting	No results posted	NCT02668770 ^115^
	Tilsotolimod (IMO-2125)	Ipilimumab	I/II	Completed	n = 35 response-evaluable patients 12 SD, 23 PD	NCT003052205 ^116^
	SD-101	Nivolumab	I	Completed	No results posted	NCT04050085
	TLR9 (CpG)	Nivolumab, irreversible electroporation	I	Recruiting	No results posted	NCT04612530
	CMP-001 (vidutolimod)	INCAGN01949	I/II	Recruiting	No results posted	NCT04387071
	TAC-001		I/II	Terminated	Safe and tolerable. No formal response data reported Preliminary efficacy reported 4 of 18 patients still receiving treatment. Terminated because of study drug not available	NCT05399654
TLR	Decoy20		I	Recruiting	N = 11 Biomarker analysis demonstrated immune activation Stable disease in one patient	NCT05651022
STING	MIW815 (ADU-S100)	Spartalizumab	Ib	Terminated	n = 67 evaluable patients (group A—solid tumor cohort) ORR = 10.4% (one CR, eight PR, 11 SD) DCR = 29.9%; mPFS = 1.9 months PFS at 6 months = 23.8% PFS at 12 months = 8.1%	NCT03172936 ^117^
		Ipilimumab	I	Terminated	No clinical benefit	NCT02675439
	BI 1387446	Ezabenlimab	Ib	Completed	Combination well-tolerated n = 26 evaluable patients (arm A [BI1387446 + ezabenlimab]) Best response was stable disease = 46.2% n = 15 evaluable (arm B [ezabenlimab alone]) Best response was stable disease = 53.3%	NCT04147234 ^118^
	IMSA101	Anti-PD1 or anti-PDL1	I/II	Completed	Combination well-tolerated Preliminary efficacy signal→ One PR reported in refractory uveal melanoma	NCT04020185 ^119^
	SYNB1891	Atezolizumab	I	Terminated	Combination well-tolerated Treatment associated with upregulation of IFN-stimulated genes, cytokines, and T-cell responses Stable disease observed in 4 of 24 (16.6%) of patients refractory to previous PD-(L)1 therapy	NCT04167137 ^120^
	BMS-986301	Nivolumab, ipilimumab	I	Completed	No results posted at this time	NCT03956680
	E7766		I	Completed	No results posted at this time	NCT04144140
	GSK3745417	Dostarlimab	I	Active, not recruiting	No results posted at this time. GSK removed drug from pipeline	NCT03843359
	SB11285	Atezolizumab	Ia/Ib	Active, not recruiting	No results posted at this time	NCT04096638
	Dazostinag (TAK-676)	Pembrolizumab	I/II	Recruiting	Single-agent dazostinag achieved a >3.5-fold induction of a 24-gene STING signature score At a dose of 5 mg (once weekly, on days 1, 8, and 15 in 21-day cycle), with or without pembrolizumab, dazostinag induced a > two-fold increase in cytokines (IFN-γ, IP-10), and increased proliferation of peripheral Ki67^+^ CD8^+^ T cells Three clinical responses observed in patients treated with dazostinag + pembrolizumab	NCT04420884
		Pembrolizumab, radiation therapy	I	Completed	No results posted	NCT04879849
	MK2118	Pembrolizumab	I	Terminated	Terminated for corporate reasons	NCT03249792
	exoSTING (CDK-002)		I	Completed	Dose-dependent activation of the STING pathway and type I IFN induction Among eight patients, tumor shrinkage observed, but no RECIST reported	NCT04592484
	Ulevostinag (MK-1454)	With or without pembrolizumab	I	Completed	n = 25 evaluable patients Six (24%) PR (three HNSCC, one TNBC, two anaplastic thyroid carcinoma) DCR = 48%	NCT03010176 ^121^
STING/CCR2	TAK-500	Pembrolizumab	I	Recruiting	No results posted	NCT05070247 ^122,123^
CCR2	PF-04136309	GEM-nab	Ib	Terminated business-related decision	n = 21 evaluable patients ORR = 23.8%	NCT02732938 ^124^
	CCX872	FOLFIRINOX	I	Completed	n = 50 evaluable patients OS at 18 months = 29%	NCT02345408 ^125^
	PF-04136309	FOLFIRINOX	Ib	Completed	n = 47 evaluable patient (n = 39 FOLFIRINOX + PF-04136309, n = 8 FOLFIRINOX) ORR: 48.5% (FOLFIRINOX + PF-04136309) *v* 25% (FOLFIRINOX) DCR: 97% (FOLFIRINOX + PF-04136309) *v* 3% (FOLFIRINOX)	NCT01413022 ^126^
CCR2/CCR5	BMS-813160	GEM-nab	I/II	Completed	No results posted	NCT03496662
		Chemotherapy or nivolumab	1b/II	Completed	No results posted	NCT03184870 ^127^
		GVAX	I/II	Recruiting	No results posted	NCT03767582 ^128^
CSF-1	Lacnotuzumab	Spartalizumab	Ib/II	Completed	Discontinued development because of the lack of efficacy One PR; nine SD Immune-related DCR = 27% n = 30 patients with PC One PR, two SD	NCT02807844 ^129^
CSF1R	Cabiralizumab	Nivolumab	Ia/Ib	Completed	n = 31 evaluable patients ORR = 10% (three PR, one SD) DCR (at 6 months) = 13%	NCT02526017
		Nivolumab, GEM	II	Suspended	No results posted	NCT03697564
		Nivolumab, GEM-nab or FOLFOX	II	Completed	Did not meet its primary end point	NCT03336216 ^130^
		Nivolumab, SBRT	II	Terminated	Terminated PI departure from institution n = 6 patients, safety cohort Four of four (100%) with unacceptable toxicity One of four (100%) proceeded to surgical resection	NCT03599362 ^131^
	IMC-CS4 (LY3022855)	Pembrolizumab, GVAX	I^a^	Completed	No results posted	NCT03153410
	AMG 820	Pembrolizumab	Ib/II	Completed	n = 116 response-evaluable patients Three ir-PR (3%), 39 ir-SD (34%) PFS = 2.1 months OS = 5.3 months	NCT02713529 ^132^
PD-1	Nivolumab	GVAX, CRS-207	II	Completed	n = 51 GVAX + CRS-207 + nivolumab (arm 1), n = 42 GVAX + CRS-207 (arm 2); mOS = 5.88 months GVAX + CRS-207 + nivolumab mOS = 6.11 months GVAX + CRS-207	NCT02243371 ^133^
	Nivolumab	GEM-nab, carboplatin	I	Completed	n = 50 response-evaluable patients with PC ORR = 18% (one CR, eight PR, 23 SD); mPFS = 5.5 months mOS = 9.9 months	NCT02309177 ^134^
	Nivolumab and irreversible electroporation		II	Recruiting	n = 8 response-evaluable patients; mPFS = 6.8 months mOS = 18.0 months	NCT03080974 ^135^
	Pembrolizumab	GEM-nab	Ib/II	Terminated	Terminated PI no longer at site n = 11 efficacy-evaluable chemotherapy-naïve patients with PC DCR = 100% mPFS = 9.1 months mOS = 15.0 months	NCT02331251 ^136^
	Pembrolizumab	mFOLFOX6	I	Terminated	Terminated PI decided to close n = 7 efficacy-evaluable patients Two (28.6%) PR, four (57.1%) SD, six (14.3%) PD	NCT02268825 ^137,138^
	Nivolumab	Ipilimumab, RT	II	Recruiting	RT, ipilimumab, and nivolumab in patients with metastatic MSS CRC (n = 40) and PC (n = 25) DCR = 37% (10 of 27; 95% CI, 19 to 58) in CRC DCR = 29% (5 of 17; 95% CI, 10 to 56) in PC ORR = 18% (3 of 17; 95% CI, 4 to 43) in PC One CRC and one PC case had a CR Deconvolution of immune subsets in RNA-seq data using immune cell transcriptional signatures revealed resting NK cells as being statistically higher in responders *v* nonresponders	NCT03104439 ^139^
	Nivolumab	Ipilimumab, RT	II	Active, not recruiting	N = 30; mPFS = 2.2 months (95% CI, 1.5 to 2.6) mOS = 2.8 months (95% CI, 2.1 to 5.2)	NCT04361162 ^140^
	Pembrolizumab		II	Completed	All patients, mPFS = 4.1 months (95% CI, 2.4 to 4.9 months) mOS = 23.5 months (95% CI, 13.5 to not reached) ORR = 34.3% (95% CI, 28.3 to 40.8) Among 22 patients with PC ORR = 18.2 (5.2 to 40.3) mPFS = 2.1 (1.9 to 3.4) mOS = 4.0 (2.1 to 9.8)	NCT02628067 ^141^
	Nivolumab	Ipilimumab, SBRT	II	Completed	N = 84 patients,→ n = 41 SBRT + nivolumab, and n = 43 SBRT + nivolumab + ipilimumab CBR = 17.1% (8.0 to 30.6) for SBRT/nivolumab CBR = 37.2% (24.0 to 52.1) for SBRT/nivolumab/ipilimumab One PR with SBRT + nivolumab Six PR with SBRT + nivolumab + ipilimumab with a median duration of response of 5.4 months (4.2 to not reached)	NCT02866383 ^142^
	Tislelizumab BGB-A317	BGB-A333	I	Terminated	n = 12 efficacy-evaluable patients (phase II) ORR = 41.7% (four CRs, one PR), mPFS = 6.1 months	NCT03379259 ^143^
PDL1	Durvalumab	Tremelimumab ± GEM-nab	II	Active, not recruiting	N = 180 patients (n = 119, GEM-nab, durvalumab + tremelimumab *v* n = 61 GEM-nab); mPFS = 5.5 months *v* 5.4 months mOS = 9.8 months *v* 8.8 months	NCT02879318 ^144^
	Durvalumab	Tremelimumab, SBRT	I	Completed	N = 59 Cohort A1 = durvalumab + SBRT 8 Gy (n = 14) Cohort A2 = durvalumab + SBRT 25 Gy in five fractions (n = 10) Cohort B1 = durvalumab + tremelimumab + SBRT 8 Gy (n = 19) Cohort B2 = durvalumab + tremelimumab + SBRT 25 Gy in five fractions ORR = 5.1% (all cohorts) Cohort A1→ mPFS = 1.7 months (0.8-2.0), mOS = 3.3 months (1.2-6.6) Cohort A2→ mPFS = 2.5 months (0.1-3.7), mOS = 9.0 months (0.5-18.4) Cohort B1→ mPFS = 0.9 months (0.7-2.1), mOS = 2.1 months (1.1-4.3) Cohort B2→ mPFS = 2.3 months (2.9-9.3), mOS = 4.2 months (2.9-9.3) NOTE: Historically, second-line chemotherapy→ mPFS of 1.8 to 3.1 months and mOS = 4.5 to 10.1 months Paired sample analyses show a nonsignificant increase in CD3- and CD8-positive cells for individual patients	NCT02311361 ^145^
	Durvalumab	Tremelimumab	II	Completed	ORR = 3.1% durvalumab + tremelimumab	NCT02558894 ^146^
LA-4	Ipilimumab (MDX-010)	GEM	I	Completed	n = 21 response-evaluable patients ORR = 14% (3 of 21) mPFS = 2.78 months mOS = 6.90 months	NCT01473940 ^147^
		GVAX	Ib	Completed	n = 12, ipilimumab alone *v* n = 13 GVAX + ipilimumab mOS: 3.6 months of ipilimumab *v* 5.7 months of GVAX + ipilimumab (*P* = .072)	NCT00836407 ^148^
			II	Completed	n = 27 response-evaluable patients No responses	NCT00112580 ^149^
	Tremelimumab (CP-675,206)		II	Completed	n = 20 efficacy-evaluable patients with mPC ORR = 0% mOS = 4 months	NCT02527434 ^150^
		GEM	I	Completed	n = 28 response-evaluable patients; mOS = 7.4 months	NCT00556023 ^151^
	Zalifrelimab	GEM-nab	I/II	Recruiting	No results posted	NCT04827953
TIGIT	Tiragolumab	Atezolizumab	I	Completed	Tiragolumab + atezolizumab was well tolerated in three Japanese patients with advanced/metastatic solid tumors One patient with PC with three previous lines of therapy achieved SD by day 42, but disease progressed. Censored PFS 1.4 months	jRCT2080224926^152^
		Atezolizumab with or without GEM-nab	I/II	Active, not recruiting	No results posted	NCT03193190

Abbreviations: ATRA, all-trans retinoic acid; CA, cancer antigen; CBR, clinical benefit rate; CR, complete response; CRC, colorectal cancer; CTLA-4, cytotoxic T lymphocyte antigen-4; DC, dendritic cell; DCR, disease control rate; DFS, disease-free survival; DLT, dose-limiting toxicity; DW, diffusion-weighted; FOLFIRINOX, folinic acid (leucovorin), fluorouracil (5-FU), irinotecan, and oxaliplatin; FU, fluorouracil; GEM, gemcitabine; GVAX, granulocyte-macrophage colony-stimulating factor secreting allogenic vaccine; HA, hyaluronic acid; HCC, hepatocellular carcinoma; HER, human epidermal growth factor; HIF, hypoxia-inducible factor; HNSCC, head and neck squamous cell carcinoma; HR, hazard ratio; IFN, interferon; IL, interleukin; mDoR, median duration of response; mOS, median overall survival; mPFS, median progression-free survival; MRI, magnetic resonance imaging; NALIRIFOX, nanoliposomal irinotecan (nal-IRI), 5-FU, leucovorin, and oxaliplatin; NSCLC, non–small cell lung cancer; ORR, objective response rate; OS, overall survival; PC, pancreatic cancer; PD, progressive disease; PFS, progression-free survival; PR, partial response; PSC, pancreatic stellate cell; RT, radiotherapy; SBRT, stereotactic body radiation therapy; SD, stable disease; TGF, transforming growth factor; TIGIT, T-cell immunoreceptor with immunoglobulin and ITIM domains; TLR, toll-like receptor; TNBC, triple-negative breast cancer; VEGF, vascular endothelial growth factor.

^a^ Drug development halted.

Inhibition of FAK-mediated integrin signaling also decreases PDAC cell survival, partially alleviating immune suppression from tumors, but preferentially drives an iCAF phenotype.^155,156^ In PDAC trials, FAK inhibitor in combination with GEM or the mitogen-activated protein kinase inhibitor, trametinib, showed little benefit.^87,88^ Immune cell profiling of pre- and post-treatment tumor samples from patients treated sequentially with GEM-nab followed by pembrolizumab and FAK inhibitor (defactinib) showed an increase in infiltrating CD8^+^ T cells.^157^ Notably, increases in CXCR4^+^ cells suggest that additional inhibition of CXCR4/CXCL12 signaling may bolster this strategy.^68^ Similarly, transcriptomic profiling of tumors from patients with locally advanced PDAC treated with defactinib and stereotactic body radiation therapy (SBRT) showed an increase in interferon (IFN)-α, IFN-β, and TNF-α pathway activity in CAF populations. This persistence of iCAFs in the TME after FAK inhibitor again suggests the importance of combining iCAF and myCAF inhibitions. Preclinically, IL-1 and IL-6 inhibition reduces JAK/STAT3 signaling and iCAF activity, decreases tumor growth, and boosts responses to anti–PD-1.^158^ Treatment with the IL-1R antagonist, anakinra, improved survival and bolstered GEM treatment.^159,160^ Solely targeting iCAF activation through the JAK pathway is not a promising strategy as treatment of patients with mPDAC with JAK inhibitor, ruxolitinib, and capecitabine (fluorouracil) showed no improvement in OS.^96^

NRG1 is another emerging TME target to consider in treatment combination strategy. Secreted by PDAC cells and CAFs, NRG1 activates human epidermal growth factor (HER) signaling in tumor cells, and higher NRG1 and HER3 are linked with poorer survival outcomes.^161^ Current therapeutic strategies primarily focus on targeting HER3, the fusion partner of NRG1, rather than NRG1 itself. Targeting the NRG1/HER3 axis may be important for breakthrough KRAS^G12D^ inhibitors as CAF-derived NRG1 activation of HER2 and HER3 allows tumors to bypass MRTX1133 sensitivity and inhibition of HER2/3 or NRG1 in human and mouse PDAC synergized with MRTX1133.^162,163^ As the development of KRAS mutant inhibitors gains momentum, translation of these preclinical findings may be realized in trials targeting NRG1/HER3 signaling.^55^

### Inhibiting Angiogenesis

Preclinical PDAC studies have assessed a variety of antiangiogenic agents, including tyrosine kinase inhibitors that inhibit oncogenic VEGF, platelet-derived growth factor, epidermal growth factor (EGF) and FGF pathways, or selective VEGFR2 blocking antibody.^164^ Despite decreasing microvascular density and increasing antitumor responses in preclinical PDAC models, trials evaluating GEM in combination with nintedanib, sorafenib, axitinib, or bevacizumab have not improved outcomes for patients with PDAC.^104-106,165,166^ Compensatory mechanisms activating MAPK/phosphoinositide 3-kinase (PI3K) signaling by other receptor tyrosine kinases may explain these failures. In a mouse model of islet cell carcinoma, anti-VEGFR2 treatment was dichotomized into early- versus late-stage response.^167^ Initial regions of hypoxia induced by VEGFR2 blockade were circumvented by tumor cell expression of other angiogenic factors such as FGF. While FGF inhibition may curb this proangiogenic rewiring, it is likely that further resistance would occur if similarly translated to patients. Rather, combining hypoxic response inhibition with VEGF inhibition to prevent hypoxia-mediated proangiogenic signaling may provide greater response in angiogenesis inhibition.

### Inhibiting Hypoxic Responses

Under tumor hypoxic conditions, activation of hypoxia-inducible factor (HIF)-1 promotes metabolism, oxidative stress, chemoresistance, and angiogenesis, with high HIF-1α linked to poor OS.^168-170^ In vitro silencing of HIF-1α under hypoxic conditions increases apoptosis and GEM sensitivity in PDAC cells.^171^ In immunocompetent PDAC mice, treatment with GEM and the HIF-1α inhibitor, PX-478, decreased tumor growth via induction of CTL-facilitated cell death.^172^ Furthermore, hypoxia and HIF activation in CAFs increase tumor progression. Specifically, HIF-1α increases the presence of iCAFs, whereas HIF-2α activation supports tumor growth via recruitment of M2 macrophages and T_reg_ cells.^173,174^ Together, this highlights the dynamic role of HIF-1α in PDAC initiation versus progression. Beyond inhibiting HIF pathways, the hypoxic PDAC TME can be exploited using prodrugs, such as evofosfamide, which in combination with GEM increased OS and PFS of patients with mPDAC.^107^ Altogether, exploiting hypoxic signaling in PDAC tumors may increase therapeutic benefits in an immunologically dependent manner.

### Activating Antitumor Immune Responses

**FIG 2.**
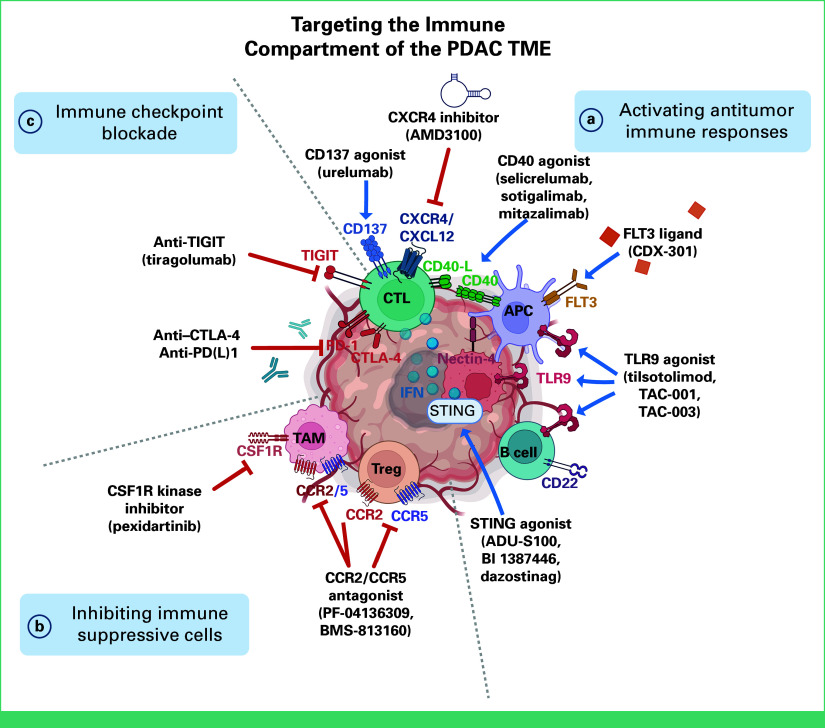
Targeting the immune compartment of the PDAC TME. A summary of the current immune-targeting therapies in PDAC including (a) activating antitumor immune responses in CTLs with CD137 agonist, activating APC activity with CD40 agonist, FLT3 ligand, activating APCs and B cells with TLR9 agonist, and stimulating tumor cells with STING agonist to release more IFNs that activate CTLs; (b) inhibiting immune suppressive T_reg_ cells and TAMs with CCR2/CCR5 antagonist and inhibiting TAMs with CSF1R kinase inhibitor; (c) preventing CTL exhaustion with immune checkpoint blockades of CTLA-4, PD-1/PD-L1, TIGIT. APC, antigen-presenting cell; CCR2, C-C motif chemokine receptor 2; CCR5, C-C chemokine receptor 5; CSF1R, colony-stimulating factor-1 receptor; CTL, cytotoxic T-lymphocyte; CTLA-4, cytotoxic T-lymphocyte–associated protein 4; FLT3, FMS-related receptor tyrosine kinase 3; IFN, interferon; PDAC, pancreatic adenocarcinoma; STING, stimulator of interferon genes; TAM, tumor-associated macrophage; TIGIT, T-cell immunoreceptor with immunoglobulin and ITIM domains; TLR9, toll-like receptor 9; TME, tumor microenvironment; T_reg_ cell, regulatory T cell.

Directly activating costimulatory receptors on CTLs is one strategy to boost antitumor immunity (Fig 2). The costimulatory receptor CD137 promotes CTL proliferation and activation, and the CD137 agonist urelumab effectively expanded CTLs in tumors ex vivo.^175,176^ In patients with resectable PDAC, anti–PD-1 and anti-CD137 agonism combined with a granulocyte-macrophage colony-stimulating factor secreting allogenic vaccine (GVAX) increased CTL infiltration and showed trending improvement in disease-free survival and OS.^113^ Although the increase did not reach statistical significance because of small sample size, the activation of CTLs with CD137 shows promises and warrants further investigation.

CD40 is a stimulatory receptor found on APCs, and its activation using anti-CD40 agonists (aCD40) enhances myeloid and DC responses and recruits CTLs and Th1 cells into the TME of PDAC mouse models.^177,178^ In combination with GEM, aCD40 promotes macrophage infiltration, stromal depletion, and tumor regression.^179^ In patients with advanced PDAC, GEM and aCD40, selicrelumab, increased immune activation, without clinically meaningful responses.^110,180^ Sotigalimab also had limited clinical benefit as a first-line treatment in combination with GEM-nab and/or nivolumab in patients with mPDAC.^181^ By contrast, first-line treatment of patients with mPDAC with the aCD40, mitazalimab, and mFOLFIRINOX achieved a nearly 21% increase in objective response rate.^111^ A look at the immunometabolism underlying CD40-mediated activation shows that epigenetic reprogramming of CD40-activated macrophages requires glutamine and fatty acid metabolism and abrogation of these metabolic pathways eliminates aCD40-induced antitumor responses.^182^ Approaches to strengthen aCD40 responses include an antifibroblast activation protein (FAP)/aCD40 bispecific antibody to improve APC homing and a recombinant FMS-like tyrosine kinase (FLT)3 ligand (CDX-301) to boost DC differentiation. Similarly, coactivation of CD40 with complementary myeloid signaling pathway dectin-1, using soluble β-glucan, is under investigation in patients with mPDAC.^183^ This approach aims to drive antitumor myeloid responses that are independent of classical T-cell cytotoxicity as an alternative to conventional ICB.^184^

As part of the pattern recognition receptor family, Toll-like receptors (TLRs) comprise a diverse array of ligand-binding proteins that bridge innate and adaptive immunity and TLR agonists seek to exploit this to boost antitumor responses. Among these is TLR9, whose high expression is associated with improved OS.^185^ While results of trials evaluating TRL9 agonists (SD-101, CpG) in combination with nivolumab are still pending, the combination of a TRL9 agonist (tilsotolimod) and ipilimumab (anti–CTLA-4) yielded poor responses.^116^ These TRL9 agonists are delivered via intratumoral injection or electroporation, so physical constraints such as tumor size and density may hinder their effects. In addition, preclinical studies suggest that pan-TLR9 activation exerts different effects on the epithelial, inflammatory, and fibrogenic cell populations within the TME.^186^ Seeking to improve TRL9 delivery, TAC-001 and TAC-003 are TLR9 agonists conjugated with anti-CD22 or anti–nectin-4 antibody, respectively. TAC-001 targets activation of CD22-expressing B cells to enhance cross-presentation and activation of innate and adaptive antitumor immune responses. Similarly, TAC-003 enables systemic delivery of TRL9 by targeting tumor-expressing nectin-4 to facilitate tumor-homing TRL9 activation of immune cells.^187^ As such, approaches for systemic delivery of TRL9 agonists may overcome challenges associated with intratumoral injection and reinvigorate antitumor immunosurveillance.

The cyclic GMP-AMP synthase (cGAS)—stimulator of interferon genes (STING)—pathway activates antitumor immune responses in innate immune cells via type I IFN release in response to cytosolic DNA that often accumulates in tumor cells.^188^ In both in vitro and in vivo PDAC studies, STING activation promotes myeloid cell maturation and T cell infiltration, expansion, and priming.^189^ Unfortunately, trials evaluating STING agonists (ADU-S100, ulevostinag) in combination with systemic anti-PD1 (spartalizumab or pembrolizumab) or anti–CTLA-4 (ipilimumab) have yielded poor efficacy signals.^117,190^ Next-generation STING agonists (BI1387446, dazostinag) have sought to improve upon stability and enable systemic delivery. BI1387446 is being evaluated alone and in combination with a novel anti–PD-1 inhibitor, ezabenlimab,^118^ with preliminary clinical assessments showing stable disease as the best responses achieved to date (ClinicalTrials.gov identifier: NCT04147234). In the absence of major clinical breakthroughs in this arena, ongoing development of STING agonists suggests continuous enthusiasm toward exploiting this pathway.

CXCR4/CXCL12 signaling is another key pathway that inhibits T-cell activation and tumor progression.^191^ In heavily treated patients with advanced colorectal cancer or mPDAC, treatment with olaptesed, an RNA aptamer that binds to CXCL12, markedly increased the proportion of patients with stable disease when given in combination with pembrolizumab.^70^ Similarly, in patients with mPDAC that progressed from GEM, the CXCR4 inhibitor BL-8040, in combination with pembrolizumab and chemotherapy, achieved a median OS (mOS) of 7.5 months, a slight increase from the historical mOS of 6.1 months with chemotherapy alone.

### Inhibiting the Infiltration of Immune Suppressive Cells

The PDAC TME is immunosuppressive because of not only the dense stroma that restricts the infiltration of antitumor immune cells but also the high presence of immune suppressive cells.^192^ Thus, strategies have sought to curb the infiltration of T_reg_ cells, TAMs, and immune suppressive myeloid subtypes. In PDAC animal models, inhibition of the CCR5/CCL5 axis prevents T_reg_ recruitment, resulting in decreased tumor progression while enhancing antitumor immunity.^193^ The clinical relevancy of these observations is being tested in a phase I/II trial of patients with locally advanced PDAC treated with the combination of a CCR2/CCR5 dual antagonist and an anti–PD-1 antibody, with or without GVAX.^128^ Another CCR2 inhibitor, PF-04136309, reduced TAM infiltration and decreased tumor growth and liver metastasis.^194,195^ These findings led to a phase Ib clinical trial in patients with borderline or locally advanced PDAC, where patients who received FOLFIRINOX and PF-04136309 had reduced tumor-infiltrating CCR2^+^ monocytes and achieved a 49% clinical response rate.^126^ Other ongoing trials are evaluating the dual CCR2/CCR5 antagonist, BMS-813160, in combination with chemotherapy alone (ClinicalTrials.gov identifier: NCT03496662) and/or with nivolumab (ClinicalTrials.gov identifier: NCT03184870) in patients with mPDAC.^127^

Inhibiting colony-stimulating factor-1 receptor (CSF1R) signaling is another approach to deplete TAMs and improve ICB responses.^197^ In PDAC mouse models, the CSF1R kinase inhibitor, pexidartinib, decreased TAM infiltration and reprogrammed the TME to support T-cell antitumor immunity. Mouse tumor volumes with pexidartinib and PD-1 or CTLA-4 ICB in combination with GEM were reduced by approximately 85%.^198^ This effect appears to be CTL-dependent as addition of anti-CD38 neutralizing antibodies reverses the tumor growth inhibition observed with pexidartinib and GEM.^199^ However, the combination pexidartinib and anti–PD-L1 (durvalumab) lacked clinical activity in patients with advanced PDAC and colorectal cancer.^200^ Preliminary findings from this trial suggest that pexidartinib reduced not only the level of circulating CD14^low^CD16^high^ monocytes but also the level of peripheral DCs. Rather, multitargeting effects of pexidartinib may also inhibit FLT3 signaling that is crucial for DC differentiation and subsequent antitumor immune responses.^201^ While antibody-directed inhibition of CSF1/CSF1R signaling may overcome these deleterious off-targeting effects, trials exploring anti-CSF1 (lacnotuzumab) or anti-CSF1R antibodies (cabiralizumab, AMG 820) have yielded insufficient antitumor activity.^129,131,202^ Findings from these trials will be important to uncover compensatory and understudied immunosuppressive cells, such as myeloid derived suppressor cells, that can be targeted in combination with CSF1/CSF1R targeting strategies.

### Immune Checkpoint Blockade

PDAC trials examining anti–CTLA-4 or anti-PD(L)1 ICBs alone or in combination have not mustered the desired responses seen in other solid tumor types. To date, benefits from ICBs are limited to <2% of all patients with PDAC that harbor microsatellite instability or a mismatch repair deficiency.^141^ While combining anti–CTLA-4 or anti-PD(L)1 ICBs with chemotherapy has yielded limited efficacy, encouraging responses have been observed among patients receiving radiation therapy.^139,142^ Patients with treatment-refractory mPDAC receiving a triplet of nivolumab, ipilimumab, and SBRT achieved a clinical benefit rate of 37.2% that lasted a median of 5.4 months.^142^ Poorer survival outcomes were noted among patients with elevated on-treatment serum levels of IL-6, IL-8, and C-reactive protein. However, the functional relevance of these cytokines remains unclear as a follow-up trial failed to show clinically meaningful benefits when incorporating IL-6R inhibitor, tocilizumab, to the combination of nivolumab, ipilimumab, and SBRT.^142^

Multiomics analysis showed additional coinhibitory molecules of CD8^+^ T cells in PDAC. One of the most highlighted axes that inhibits T-cell function is CD155/TIGIT, spurring further clinical investigation.^203^ In a cohort of 3 Japanese patients with advanced/metastatic solid tumors, the combination of atezolizumab and anti-TIGIT, tiragolumab, was well-tolerated, with one patient with heavily pretreated PDAC briefly achieving stable disease after 42 days of treatment before experiencing disease progression.^152^ A cohort of the umbrella Morpheus-PDAC trial is currently examining atezolizumab, tiragolumab, and GEM-nab as a first-line treatment (ClinicalTrials.gov identifier: NCT03193190). Even so, it is unlikely that the combined PD-1/TIGIT blockade can sufficiently elicit robust antitumor responses as preclinical mouse models show that reductions in tumor growth were only achieved after addition of aCD40 to the coblockade of TIGIT/PD-1.^203^ Another study using a neoantigen vaccine approach in a KRAS-driven mouse model linked slowing of tumor growth to increases in neoantigen-specific T cells expressing high levels of TIGIT and PD-1, whereas subsequent coblockade of TIGIT/PD-1 enhanced immune responses.^204^

## DISCUSSION

In conclusion, a highly diverse PDAC TME significantly limits the effectiveness of many therapies.^205^ Distinct substates within the TME containing iCAF and myCAF cells and numerous intermediate CAF subpopulations are indicative of dynamic shifts between these two TME states within the same patient and underscore the challenges involved in targeting the complex PDAC TME. Adding to the complexity, awareness of the cross talk that KRAS activation enables aberrant metabolic and cellular bioenergetics within tumor cells and the surrounding TME provides another potential area for therapeutic exploitation.^206^ For instance, under nutrient-deprived conditions, CAFs support cancer cell growth by providing amino acids and other essential metabolites that enable tumor cells to maintain homeostasis.^207^ A greater understanding of the metabolic cross talk between tumor cells, CAFs, and infiltrating immune cells that influences tumor progression and response to treatment may profoundly improve upon current therapeutic strategies.

On the basis of the above-reviewed studies in the field, the multifaceted complexity within the PDAC TME underscores the need for therapeutic interventions directed at inhibiting both tumor cell–intrinsic features and various tumor-promoting components within the TME. Continuous efforts to personalize cancer care require not only molecular subtyping of patient tumors but also TME profiling.^208^ As trials focus on targeting different components of the PDAC TME, we suggest that studies include more comprehensive readouts on how treatments influence the biologic behavior of tumor, immune, and stromal cells within the TME, regardless of whether patient outcomes improve. Furthermore, as clinical trials continue to rely on preclinical data, it is crucial to perform studies with the most rigorous and relevant models.^209,210^ We believe that the field is primed to leverage our growing knowledge of the PDAC TME, along with the excitement of new targeted strategies against tumor-intrinsic properties (eg, KRAS), to improve overall patient outcomes against this lethal cancer.
